# Supplementary material for: Butyrate producing colonic Clostridiales metabolise human milk oligosaccharides and cross feed on mucin via conserved pathways
Source: Nat Commun. 2020 Jul 3;11:3285. doi: 10.1038/s41467-020-17075-x (PMC7335108; doi:10.1038/s41467-020-17075-x)
Supplement: Supplementary file 1 — Supplementary Information [file 41467_2020_17075_MOESM1_ESM.pdf]

Supplementary Information for:

Butyrate producing colonic Clostridiales metabolise human milk oligosaccharides and cross feed on mucin via conserved pathways

Pichler M.J. et al.

Supplemental Information:

Supplementary Tables:

Supplementary Table 1: Human milk and blood antigen oligosaccharides studied in this work

Compound[a]	Abbreviation	Supplier	Structure[b]
Lactose	Lac	Sigma Aldrich	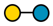
2'-Fucosyllactose	2'FL	IsoSep	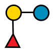
3-Fucosyllactose	3FL	IsoSep	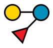
3'-Sialyllactose	3'SL	Carbosynth	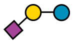
6'-Sialyllactose	6'SL	Carbosynth	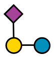
Difucosyllactose	DFL	Dextra Laboratories	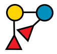
Lacto-*N*-biose	LNB	Elicityl	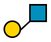
Galacto-*N*-biose	GNB	Sigma Aldrich	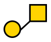
Lacto-*N*-tetraose	LNT	Elicityl<br>IsoSep	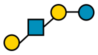
Lacto-*N*-*neo*tetraose	LN*n*T	Dextra Laboratories	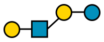
Lacto-*N*-fucopentaose I	LNFP I	Dextra Laboratories	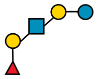
Lacto-*N*-fucopentaose II	LNFP III	Honeywell Fluka	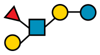
Lacto-*N*-fucopentaose III	LNFP III	Carbosynth	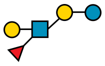
Lacto-*N*-difucohexaose I	LNDFH I	Dextra Laboratories<br>Carbosynth	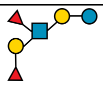
Lacto-*N*-difucohexaose II	LNDFH II	Elicityl<br>Dextra Laboratories	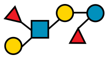
Blood group antigen H triose type 1	H triose type 1	Elicityl	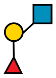
Blood group antigen A triose	A triose	Elicityl	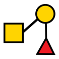
Lewis A antigen triose	Le[a] triose	Elicityl	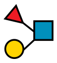
Lewis B antigen tetraose	Le[b] tetraose	Carbosynth	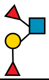

[a]All carbohydrates were > 95% pure unless otherwise stated.
[b]Glycan structures presentation according to Symbol Nomenclature for Glycans (SNFG) (https://www.ncbi.nlm.nih.gov/glycans/snfg.html)

Supplementary Table 2: Binding and thermodynamic parameters of *Rh*LNBBP determined by ITC.

Ligand	K_D (μM)	N_0	ΔH (kcal mol^{-1})	$-T\Delta S$ (kcal mol^{-1})	ΔG (kcal mol^{-1})
LNB	2.64 ± 0.39	0.77 ± 0.01	-30.37 ± 0.66	22.75	-7.62
	3.12 ± 0.48	0.94 ± 0.01	-28.14 ± 0.56	20.63	-7.51
GNB	11.11 ± 0.57	1.26 ± 0.02	-12.28 ± 0.31	5.52	-6.76
	10.92 ± 0.61	1.24 ± 0.02	-13.58 ± 0.28	6.83	-6.75
LNT	10.95 ± 0.43	0.89 ± 0.01	-19.12 ± 0.21	12.34	-6.78
	9.71 ± 0.26	0.86 ± 0.00	-19.60 ± 0.11	12.76	-6.84
Lactose	n.b.				
2'FL	n.b.				

Data are from independent duplicates and binding parameters are reported with the error of the fit to the binding isotherm. n.b.: Affinity too low to be determined. N_0: Is the molar binding stoichiometry.

Supplementary Table 3: Binding parameters of *Ri*Le$^{a/b}$BP determined by SPR

Ligand	K_D (μM)	R_{max}	χ^2
LNB	6.63 ± 0.68	10.4	0.10
	6.91 ± 0.84	9.7	0.12
GNB	10.82 ± 0.77	10.4	0.06
	10.21 ± 0.77	8.8	0.03
Le^b tetraose	1.77 ± 0.12	25.4	0.45
	1.75 ± 0.17	24.8	0.51
Le^a triose	3.31 ± 0.46	17.3	0.32
	2.99 ± 0.38	17.6	0.47
H triose type I	11.2 ± 1.1	8.8	0.06
	11.3 ± 1.5	9.0	0.12
LNT	n.b.		
Blood group A antigen triose	n.b.		
2'FL	n.b.		
3'FL	n.b.		
LN*n*T	n.b.		
Lactose	n.b.		

Data are from independent duplicates and binding parameters are reported with the standard error of the fit to a one binding site model. n.b.: Indicates low affinity to ligand precluding determination of binding parameters. R_{max} and χ^2: Denote the maximum binding level and the statistical goodness of the fit to a one binding site model, respectively.

Supplementary Table 4. Kinetic parameters of *Rh*Lnb136, *Ri*Le$^{a/b}$136 and *Er*Lnb136

Substrate	Enzyme	K_M (mM)	k_{cat} (s^{-1})	k_{cat}/K_M (s^{-1} mM^{-1})	specific activity[a] (U mg^{-1})
LNT	*Rh*Lnb136	1.45 ± 0.05	86 ± 1	59.3	58.5 ± 0.58
LNT	*Ri*Le$^{a/b}$136	-	-	-	0.21 ± 0.00
LNT	*Er*Lnb136	0.68 ± 0.07	160 ± 7	235.3	
LNT	*Er*Lnb136 Y145A	n.d.	n.d.	48	

[a] specific activity determined towards 3.5 mM LNT. n.d.: Lack of curvature of the Michaelis Menten plot preclude determination of kinetic parameters. Data are means of triplicates with standard deviation (SD).

Supplementary Table 5. Specific activities of *Rh*GLnbp112 *and Ri*GLnbp112

Substrate	***Rh*GLnbp112** (U mg^{-1})	***Ri*GLnbp112** (U mg^{-1})
LNB	12.2 ± 0.5	22.6 ± 0.2
GNB	9.6 ± 0.1	16.9 ± 0.4

Data are means of triplicates with standard deviation (SD). Specific activities determined towards 2 mM LNB or GNB.

Supplementary Table 6: Crystallographic data collection and refinement statistics.

	*Er*Lnb136 Se-Met	*Er*Lnb136 Native
**PDB ID**	6KQS	6KQT
**Data collection**[a]		
Beamline	SLS X06DA	KEK-PF BL5A
Wavelength (Å)	0.978	1.000
Space group	$P3_121$	$P3_121$
Unit cell (Å)	$a = b = 132.7$, $c = 82.5$	$a = b = 132.3$, $c = 82.2$
Resolution (Å)	45.75–1.40 (1.42–1.40)	50.0–2.00 (2.03–2.00)
R_{merge}	0.145 (1.909)	0.233 (1.065)
Number of observations	3,288,573 (155,651)	556,706
Unique reflections	153,888 (8,031)	56,318 (2,757)
Mean $I/\sigma(I)$	12.2 (1.7)	13.4 (3.0)
CC (1/2)	0.999 (0.728)	0.980 (0.824)
Completeness (%)	100.0 (100.0)	100.0 (100.0)
Multiplicity	20.1 (19.4)	9.9 (9.6)
Anomalous completeness (%)	100.0 (100.0)	–
Anomalous multiplicity	10.2 (9.8)	–
**Refinement**		
Resolution	47.20–1.40	47.04–2.00
No. of reflections	155,798	53,440
R factor/R_{free} (%)	14.8 (17.2)	14.3 (18.1)
No. of atoms	5,920	5,787
No. of solvents	835 (water), 1 (glycerol)	706 (water), 1 (triethylene glycol), 1 (Na^+)
RMSD from ideal values		
Bond lengths (Å)	0.016	0.011
Bond angles (°)	1.975	1.63
Ramachandran plot (%)		
Favored	95.9	95.8
Allowed	4.1	4.2
Outlier	0	0

[a]Values in parentheses are for the highest resolution shell.

Supplementary Table 7: Summary of structural similarity Dali server search of *Er*Lnb136.

Protein	Source organism	PDB (chain)	Z score	RMSD (Å)	N_{align}[a]	$\%_{seq}$[b]
***Er*Lnb136$_{I}$ (residues 7-224)**						
SurA-like putative peptidyl-prolyl cis-trans isomerase	*Helicobacter pylori*	5EZ1 (A)	7	3.2	70	19 (6)
Hypothetical protein LIC12922	*Leptospira interrogans*	3NRK (A)	5.8	4.8	105	10 (5)
***Er*Lnb136$_{II}$ (residues 242-663)**						
LnbX (GH136)	*Bifidobacterium longum*	5QQC (H)	50.3	1.4	416	44 (43)
α-L-fucosidase BT_1002 (GH141)	*Bacteroides thetaiotaomicron*	5MQP (F)	32	3.1	312	16 (12)

Data were obtained using Dali server.
[a]Number of aligned residues
[b]Sequence identity of aligned residues and the corresponding overall (global) sequence identity showed in parenthesis

Supplementary Table 8. Primers for cloning and mutagenesis.

Locus tag/Primer name	Accession[a]	Designation	Orientation	Sequence (5'->3')
RHOM_04115_FP	G2T0V1	*Rh*Lnb136$_{II}$	Forward	AGGAGATATACCATGGATGACAGGCTCATACAGGAC
RHOM_04115_RP	G2T0V1	*Rh*Lnb136$_{II}$	Reverse	GGTGGTGGTGCTCGAGGCCCAACGGAATAATCGTATTATCC
RHOM_04110_FP	G2T0V0	*Rh*Lnb136$_{I}$	Forward	AGGAGATATACCATGGATGAATCGGAAATATTGTCTGGAT
RHOM_04110 _RP	G2T0V0	*Rh*Lnb136$_{I}$	Reverse	GGTGGTGGTGCTCGAGGCCCGCCCGGTTTTCTGA
RHOM_04120_FP	G2T0V2	*Rh*GLnbp112	Forward	AGGAGATATACCATGGATGACTTTAAAAGAGGGACGTG
RHOM_04120_RP	G2T0V2	*Rh*GLnbp112	Reverse	GGTGGTGGTGCTCGAGGCCAATGTTATACCATTTAATCTCG
RHOM_04095 (AA36-454)_FP	G2T0U7	*Rh*LNBBP	Forward	TTTCAGGGCGCCATGGGTGCAGCTGAAACCAGCC
RHOM_04095 (AA36-454)_RP	G2T0U7	*Rh*LNBBP	Reverse	GACGGAGCTCGAATTCTTATTCACTAATGTTAAATTCAAC
ROSEINA2194_01898 (AA26-861)_FP	C0FT31	*Ri*Le$^{a/b}{}_{II}$136	Forward	TTTCAGGGCGCCATGGGTAATGCAGGGACAACCT
ROSEINA2194_01898 (AA26-861)_RP	C0FT31	*Ri*Le$^{a/b}{}_{II}$136	Reverse	GACGGAGCTCGAATTCTTATCTTCTGTAAAGCTCAAATTCT
ROSEINA2194_01899 (AA36-340)_FP	C0FT32	*Ri*Le$^{a/b}{}_{I}$136	Forward	CAGCCATATGCTCGAGGGAGAAAATATTAAGATTTCCAAAG
ROSEINA2194_01899 (AA36-340)_RP	C0FT32	*Ri*Le$^{a/b}{}_{I}$136	Reverse	CAGCCGGATCCTCGAGCTAATTCCATTTAATCGTATCG
ROSEINA2194_01885_FP	C0FT18	*Ri*GLnbp112	Forward	TTTCAGGGCGCCATGGGTAATAAAGAACACGGTGGAAGAGT
ROSEINA2194_01885_RP	C0FT18	*Ri*GLnbp112	Reverse	GACGGAGCTCGAATTCTTAAACAGCGTACCATTTAATCTCA
ROSEINA2194_01895 (AA23-470)_FP	C0FT28	*Ri*Le$^{a/b}$BP	Forward	TTTCAGGGCGCCATGGGAAATGCAAATACATCCGCAAACAC
ROSEINA2194_01895 (AA23-470)_RP	C0FT28	*Ri*Le$^{a/b}$BP	Reverse	GACGGAGCTCGAATTCTTATTGCGCAGTTTCTGAAACCTC
ROSEINA2194_01891/01890_FP	C0FT24/C0FT23	*Ri*Fuc29	Forward	TTTCAGGGCGCCATGGGGAGGACACCCGAAGAACAGA
ROSEINA2194_01891/01890_RP	C0FT24/C0FT23	*Ri*Fuc29	Reverse	GACGGAGCTCGAATTCTTATGATTCTTGATAAACCTCAA
ROSEINA2194_01889/01888/01887_FP	C0FT22/C0FT21/C0FT20	*Ri*Fuc95	Forward	TTTCAGGGCGCCATGGGGGATTTAAGTAAATATGATATTTG
ROSEINA2194_01889/01888/01887_RP	C0FT22/C0FT21/C0FT20	*Ri*Fuc95	Reverse	GACGGAGCTCGAATTCTTATCCTGTAATTTTTGCATTTC
ROSEINA2194_02198 (AA29-975)_FP	C0FTX7	*Ri*GH98	Forward	TTTCAGGGCGCCATGGGCAAAACGGGATCAGAAT
ROSEINA2194_02198 (AA29-975)_RP	C0FTX7	*Ri*GH98	Reverse	GACGGAGCTCGAATTCTTAAACTATATCAAAATACACAT
HMPREF0373_02965_FP	U2PDT9	*Er*Lnb136	Forward	TTTCAGGGCGCCATGGGAAAATTGTGTGAAAATCAGCAGG
HMPREF0373_02965_RP	U2PDT9	*Er*Lnb136	Reverse	GACGGAGCTCGAATTCTTAAATCAGATGGATTTCATTCTCC
HMPREF0373_02965_FP	U2PDT9	*Er*Lnb136_Y145A	Forward	CGAAAACAGATCACCATGAGCCCTGGTAAACAGATCCTGG
HMPREF0373_02965_RP	U2PDT9	*Er*Lnb136_Y145A	Reverse	CCAGGATCTGTTTACCAGGGCTCATGGTGATCTGTTTTCG

[a] UniProtKB accession number

Supplementary Figures:

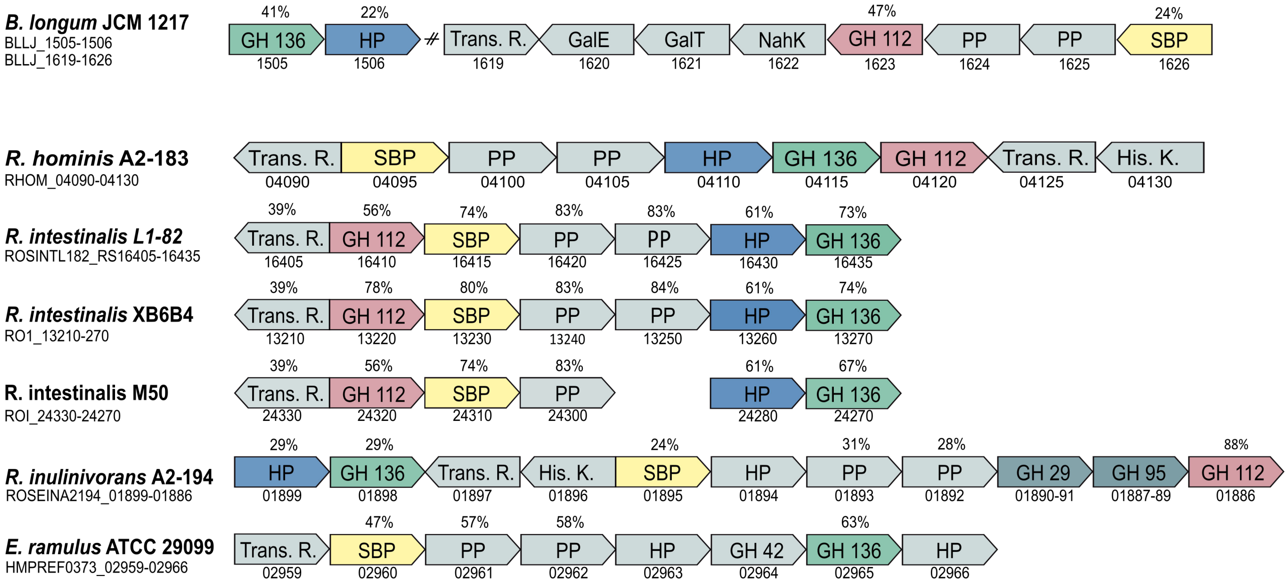

Supplementary Fig. 1: The conservation of the HMO utilization loci within *Roseburia* spp. and *Eubacterium ramulus*. The isolated loci encoding the GH136 and the LNB/GNB utilisation in *Bifidobacterium longum* subsp. *longum* JCM 1217 are included for comparison. Gene locus IDs are below the genes, which are denoted according to their protein products: UDP-glucose/GlcNAc 4-epimerase (GalE); UDP-glucose-hexose 1-phosphate uridylyl transferase (GalT); *N*-Acetylhexosamine 1-kinase (NahK); transcriptional regulator (Trans. R.); ABC transporter solute binding protein (SBP); ABC transporter permease protein (PP); Hypothetical proteins (HP), glycoside hydrolase (GH) and histidine kinase sensory protein (His. K.). Sequence identities to the *R. hominis* A2-183 corresponding homologs are above the genes. Genes encoding GH136 family members were identified via the dbCAN database.

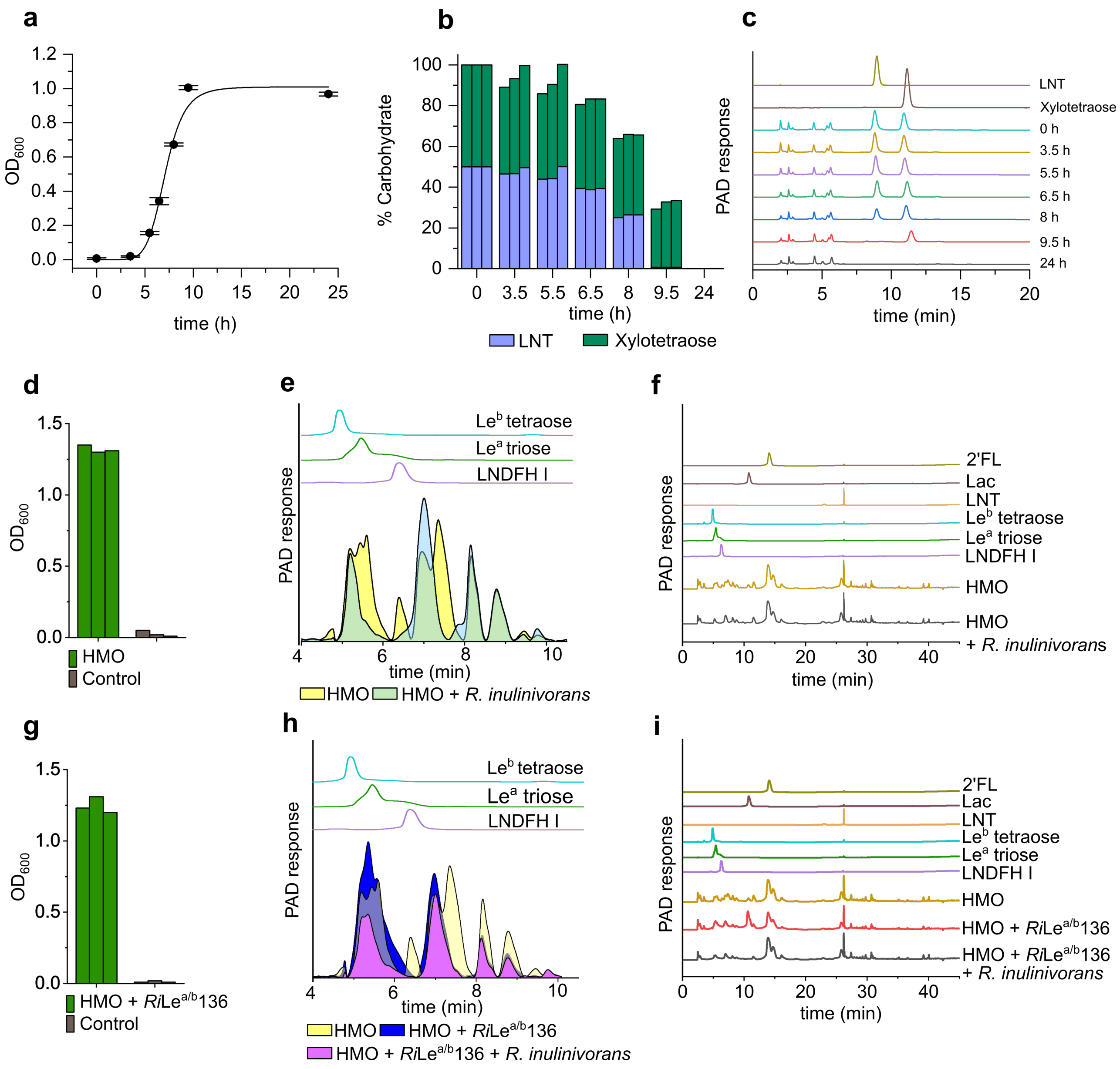

Supplementary Fig. 2: Oligosaccharide uptake profiles of *Roseburia*. (a-c), Oligosaccharide uptake of *R. hominis* during growth on an equimolar LNT and xylotetraose mixture. **(a),** Growth curve of *R. hominis* on YCFA supplemented with 0.5 % (w/v) of an equal mixture of xylotetraose and LNT. **(b),** Time course of relative percentages of xylotetraose and LNT in culture supernatants from (a) calculated based on HPAEC-PAD analyses as exemplarily represented in (c). **(c),** HPAEC-PAD chromatograms showing time course analysis of culture supernatants of *R. hominis* grown on YCFA supplemented with 0.5 % (w/v) of an equal mixture of xylotetraose and LNT. **(d-i),** HMO uptake preference of *R. inulinivorans* growth on 0.5 % purified HMOs from mother's milk. **(d)** Growth levels from *R. inulinivorans* on HMOs from mother's milk within 24h including a non-carbon source control and **(e-f)** HPAEC-PAD chromatograms (**(e)** segment 4-10 min selected from **(f)** full elution profile) showing the time course analysis of culture supernatants of *R. inulinivorans* as grown in (d). **(g)** Growth levels from *R. inulinivorans* on HMOs from mother's milk previously digested with *Ri*Le$^{a/b}$136 (0.5 µM for 18h) within 24h including a non-carbon source control and **(h-i)** HPAEC-PAD chromatograms (**(h)** segment 4-10 min selected from **(i)** full elution profile) showing the time course analysis of culture supernatants of *R. inulinivorans* as grown in (g). Observed peaks between 0 and 6 minutes (c) and observed peaks between 0 and 4 minutes (f and g) are medium components. Growth experiment (a, d and g) were performed as independent biological triplicates (n=3,) and the growth data in (a) are represented as mean values with the error bars showing the standard deviation (SD). The HPAEC-PAD analyses (b, c, e, f, h and i) were performed in triplicates (one analysis from each biological growth condition replicate) whereby all analyses yielded similar results. Source data are provided as a Source Data file labelled with the corresponding figure number and panel definition.

a *R. inulinivorans* L-fucose acid utilization locus

Locus ID	Log2 fold change HMOs/Glc	Log2 fold change Mucin/Glc	Protein	Annotation
01688				Transcriptional regulator
01689	6.64	6.64	fucI	L-fucose isomearse
01690				Hypothetical protein
01691	2.52		ABC-PP	ABC transporter, permease protein
01692	3.04	6.64	ABC-PP	ABC transporter, permease protein
01693	5.18	4.72	ABC-PP	ABC transporter, permease protein
01694			ABC-PP	ABC transporter, permease protein
01695	5.59	6.64	ABC-SBP	ABC, solute binding protein
01696	1.65	2.1		FucU transport protein
01697	0.37			Hypothetical protein
01698				Carbohydrate kinase
01699	1.15	1.92	fucK	L-fuculokinase
01700	1.87			Nucleotide binding domain ParA family protein
01701	1.74	1.71		Hypothetical protein
01702	1.46			Hypothetical protein
01703	2.58	5.2		BMC domain protein
01704				Ethanolamine/Propanediol utilization protein
01705	3.82	3.64	fucA	L-fuculophasphate aldolase
01706	2.43			Hypothetical protein
01707	3.97	3.35		Hypothetical protein
01708	3.15	3.68		CoA dependent aldehyd dehydrogenase
01709	3.83	4.12		Alcohol dehydrogenase
01710	2.77	4.24		BMC domain protein
01711	3.21	3.12		BMC domain protein
01712	2.49	3.01		BMC domain protein
01713	2.73	2.67		BMC domain protein
01714	3.39	3.64		Phosphate propanoyltransferase
01715	4.27			Ethanolamine/Propanediol utilization protein
01716	3.07	2.29		NADH dehydrogenase like subunit protein
01717	2.11	2.52		BMC domain protein
01718	2.65			Transcriptional regulator
01719	3.14	2.82		Propandiol dehydratase
01720	2.64			Pyruvate lyase

Log2- fold change: -10, 0, 10

b *R. inulinivorans N*-acetylneuraminic acid utilization locus

Locus ID	Log2 fold change HMOs/Glc	Log2 fold change Mucin/Glc	Protein	Annotation
00873				Transcriptional regulator
00874	2.11	1.32	nagB	glucosamine-6-phosphate deaminase
00875	0.28	1.65		Transcriptional regulator
00876	2.96	4.71		ABC transporter, solute binding protein
00877				Hypothetical protein
00878			ABC-PP	ABC transporter, permease protein
00879			ABC-PP	ABC transporter, permease protein
00880	2.57	5.02	nanA	N-acetylneuraminate lyase
00881	1.95	3.67	YhcH	YhcH family protein
00882	2.71	4.25	nanE	N-acetylmannosamine-6-phosphate epimerase
00883	2.89	4.65	nanK	N-acetylmannosamine kinase
00884	1.52	3.21	AE	Acetylesterase

Log2- fold change: -10, 0, 10

Supplementary Fig. 3: *R. inulinivorans* upregulated L-fucose and *N*-acetylneuraminic acid utilization loci. (a) Upregulation of L-fucose utilization cluster of *R. inulinivorans.* **(b)** Upregulation of putative *N*-acetylneuraminic acid utilization cluster of *R. inulinivorans* cells grown on purified HMOs from mother's milk and mucin, respectively, relative to glucose (Glc). **(a,b)** The heat maps depict Log2-fold changes of proteins expressed by cells grown on HMOs or mucin, respectively, relative to glucose. Locus numbers Roseina2194_xxxxx are abbreviated with the last numbers after the hyphen.

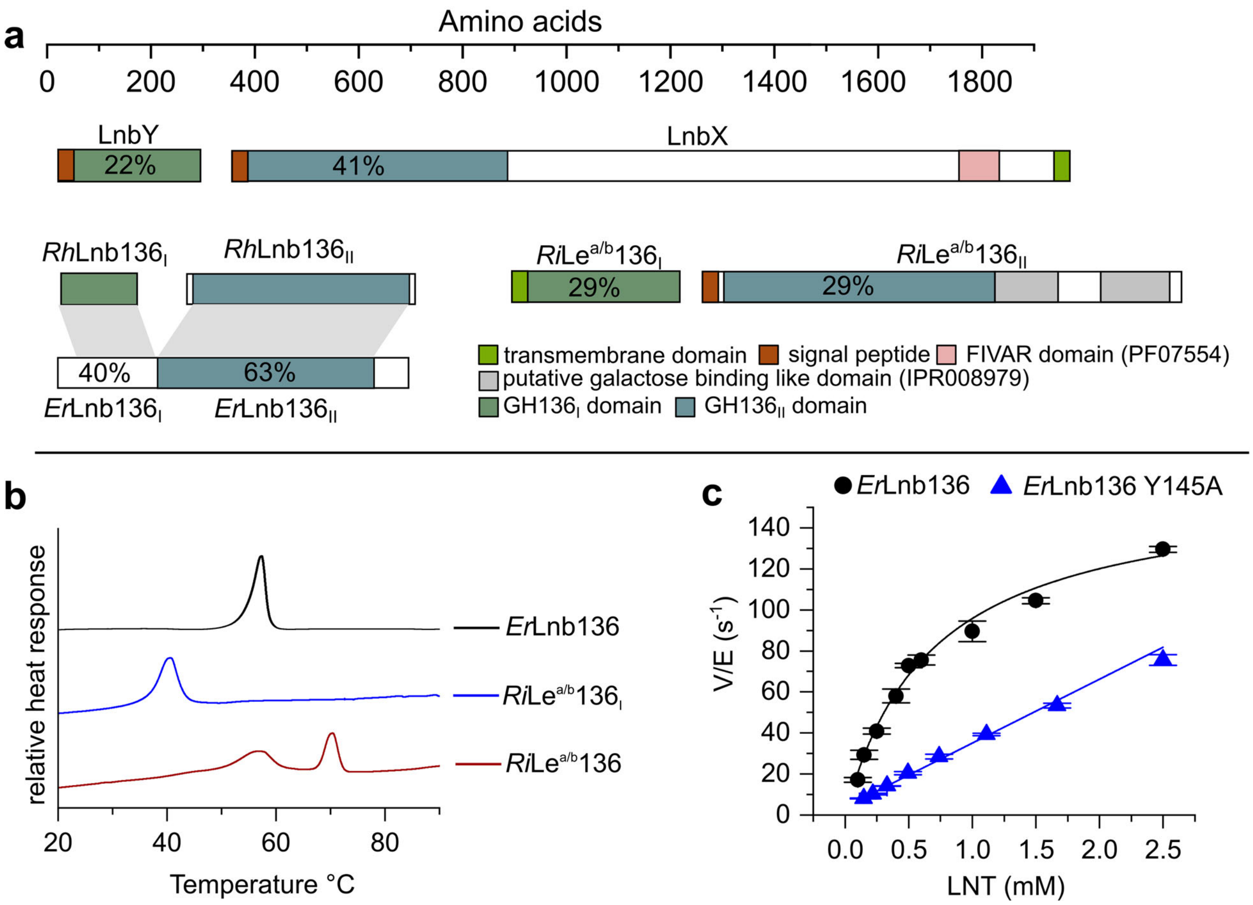

Supplementary Fig. 4: Organization, stability and functional interactions of GH136 domains. (a) Domain organization of GH136 enzymes in *B. longum* (LnbY, LnbX), *R. inulinivorans* (*Ri*), *R. hominis* (*Rh*) and *E. ramulus* (*Er*). Amino acid sequence identities to the corresponding *R. hominis* homologues are indicated within the genes. **(b)** Differential scanning calorimetry thermograms showing the unfolding of *Er*Lnb136 and of *Ri*Le$^{a/b}$136$_{I}$ and *Ri*Le$^{a/b}$136. The unfolding of the two domains of ErLNb136 appears to overlap giving rise to a single asymmetric thermal transition consistent with the cooperative unfolding of the domains. By contrast, the unfolding of *Ri*Le$^{a/b}$136 features two well resolved transitions, the first is likely attributed to the unfolding of the *Ri*Le$^{a/b}$136$_{I}$ domain while the second is likely to be attributed to the unfolding of the remaining part of the protein including the *Ri*Le$^{a/b}$136$_{II}$ domain. **(c)**, Hydrolysis kinetics of *Er*Lnb136 and the mutant *Er*Lnb136 Y145A on LNT. DSC analyses (b) were performed as duplicates whereby all analyses yielded similar results and kinetic measurements (c) were performed as triplicates. Source data are provided as a Source Data file labelled with the corresponding figure number and panel definition.

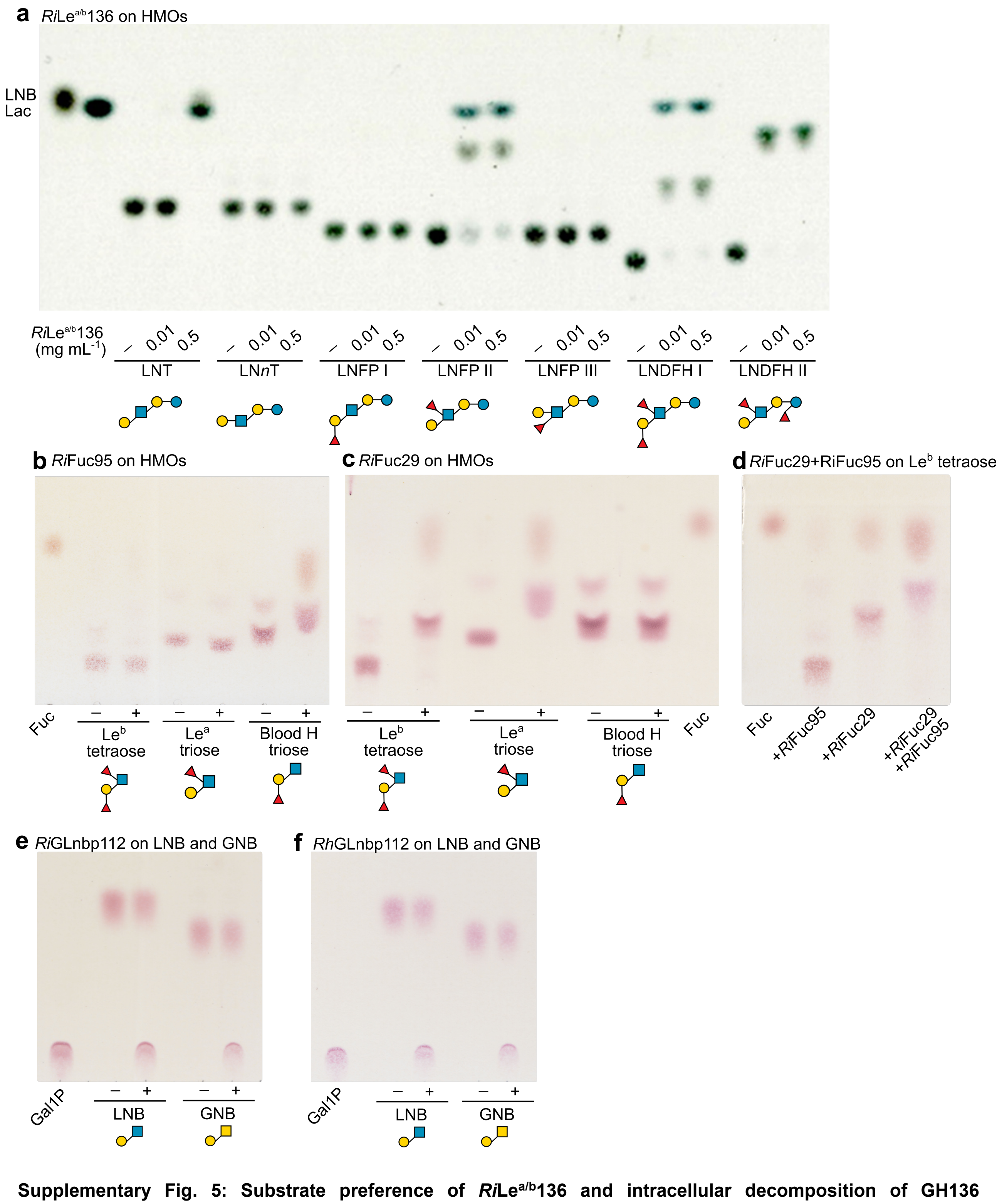

Supplementary Fig. 5: Substrate preference of *Ri*Le$^{a/b}$136 and intracellular decomposition of GH136 degradation products in *Roseburia*. **(a)**, Substrate preference of *Ri*Le$^{a/b}$136 towards HMOs; reactions with 0.01 or 0.5 mg mL^{-1} of *Ri*Le$^{a/b}$136, respectively. **(b)**, Fucosidase activity of *Ri*Fuc95 on HMOs. **(c)**, Fucosidase activity of *Ri*Fuc29 on HMOs. **(d)**, Complete defucosylation of Le^{b} tetraose by orchestral action of *Ri*Fuc29 and *Ri*Fuc95. Data show hindrance of *Ri*Fuc95 by α-(1→2)-linked L-fucose on Le^{b} tetraose. **(e)**, Phosphorylase activity of *Ri*GLnbp112. **(f)**, Phosphorylase activity of *Rh*GLnbp112. **(a-f)**, +: reactions with enzyme, -: controls without enzyme. Analyses were performed in independent duplicates whereby all analyses yielded similar results. Source data are provided as a Source Data file labelled with the corresponding figure number and panel definition.

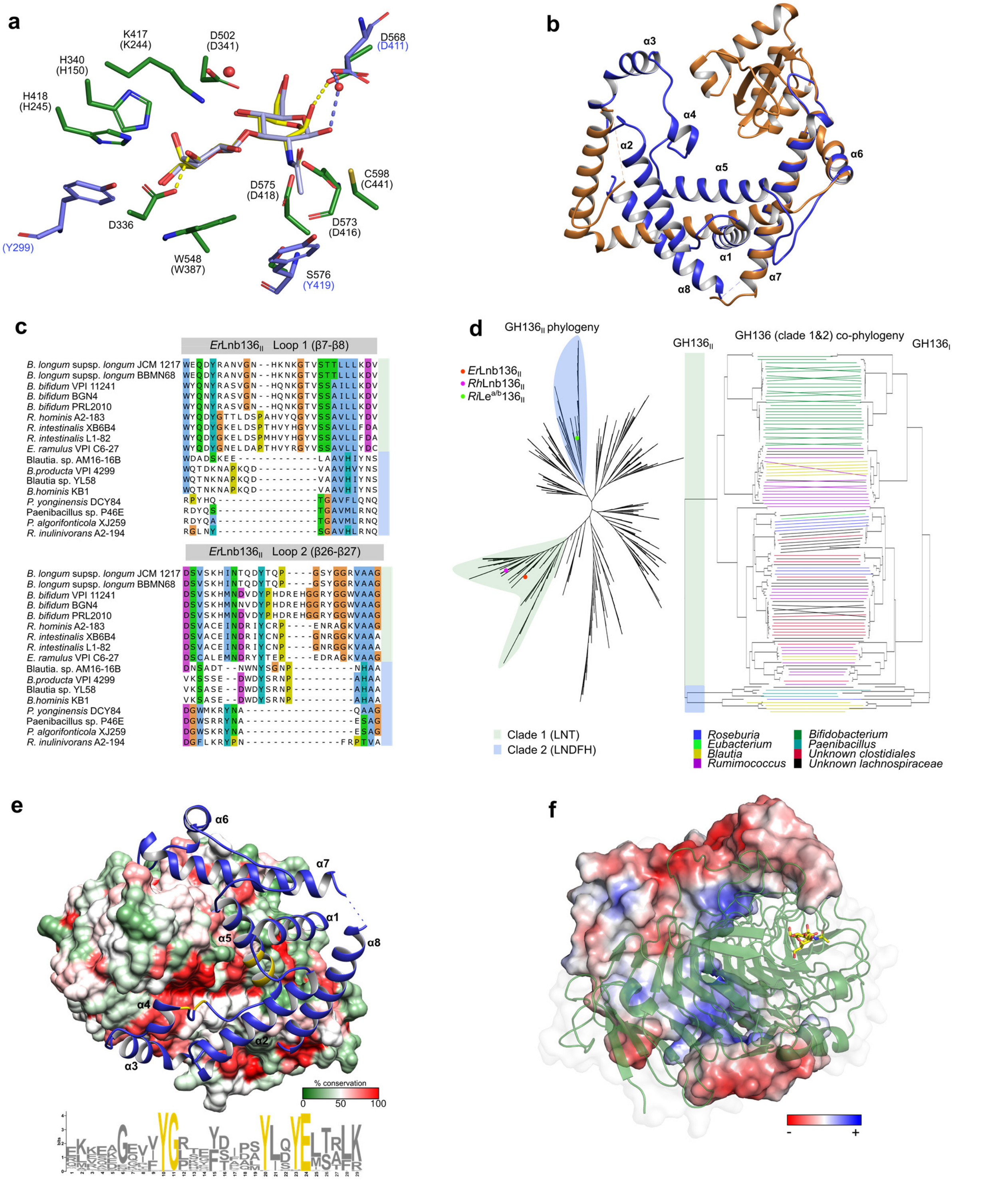

Supplementary Fig. 6: Evolution of GH136 enzymes. (a) Superimposition of LNB (yellow) bound in *Er*Lnb136 (green) and LNB (grey) in *Bl*LnbX from *B. longum* (light blue). Conserved residues are shown for *Er*Lnb136 and *Bl*LnbX (in parentheses). Residues Y299, Y419 and D411 of *Bl*LnbX that are variant compared to *Er*Lnb136 are shown in light blue to highlight differences in active site architecture and ligand binding. Water molecules are red spheres and hydrogen bonds are dashed lines in *Er*Lnb136 (yellow) and *Bl*LnbX (light blue). **(b)** Superimposition of *Er*Lnb136$_{I}$ (blue) and most related structural homolog 5EZ1 (chain A) from *Heliobacter pylori* (orange), highlighting the large differences in protein fold. **(c)** Partial amino acid sequence alignment of GH136$_{II}$ domains showing shortened loops around the active site in *R. inulinivorans* as compared to *Er*Lnb136 of *E. ramulus* **(d)** Phylogenetic tree of 985 GH136$_{II}$ sequences identified by BLASTP search of *Rh*Lnb136$_{II}$ or *Ri*Le$^{a/b}$136$_{II}$ against non-redundant database (sequences with an e-values < 10^{-10} are included). Tanglegram showing co-evolution of GH136$_{I}$ and GH136$_{II}$ domains across 117 selected sequences of GH136$_{II}$ phylogenetic tree clade 1 and clade 2. **(e)** Surface of *Er*Lnb136$_{II}$ coloured by amino acid sequence conservation across 117 sequence as presented in (d) and cartoon presentation (blue) of *Er*Lnb136$_{I}$ with conserved residues (yellow) as identified from a sequence motif generated via the MEME suite from 117 GH136$_{I}$ sequences as in (d). **(f)** Electrostatic surface of *Er*Lnb136$_{I}$ and cartoon presentation of ErLnb136$_{II}$ (green).

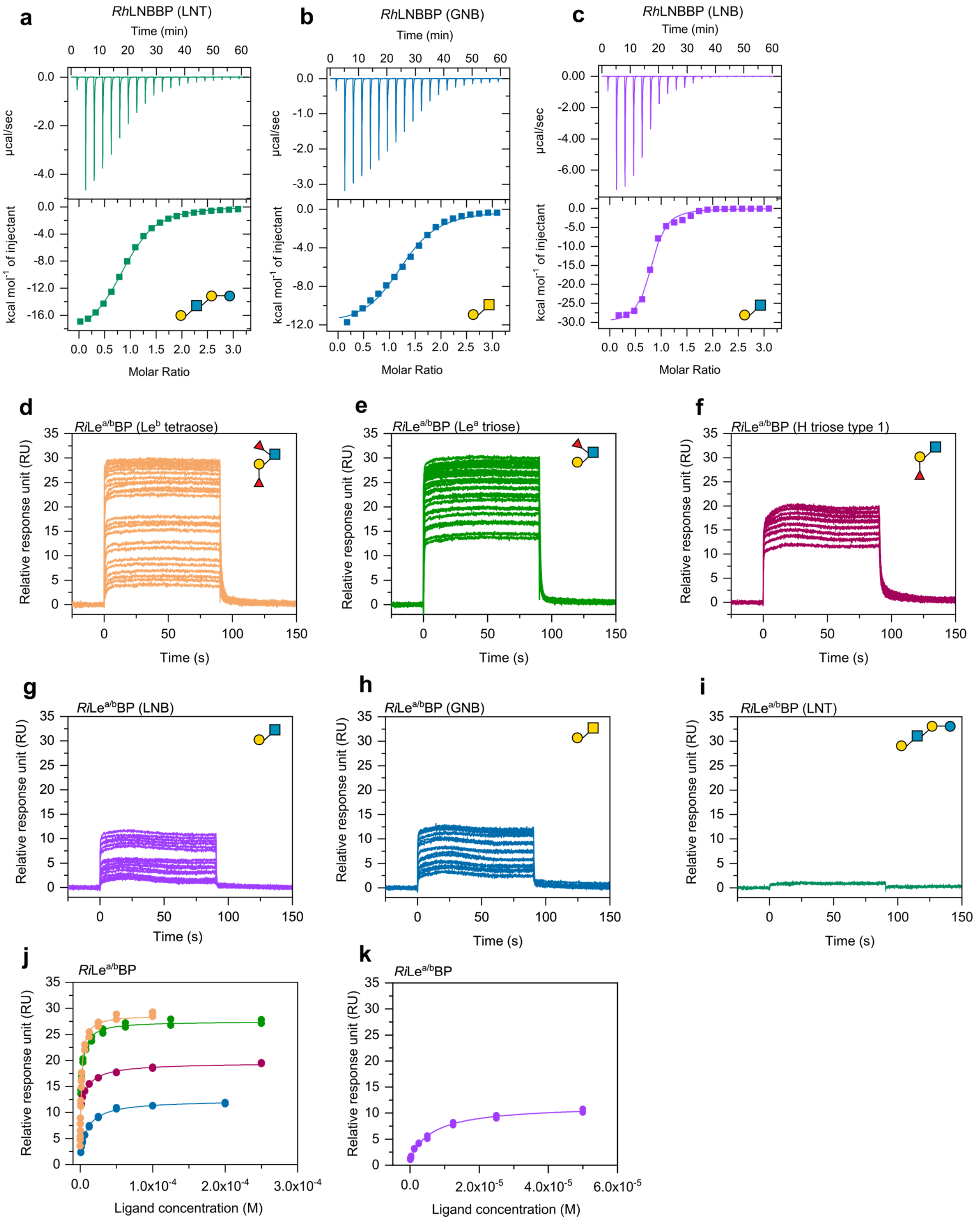

.

Supplementary Fig. 7: Binding of *Rh*LNBBP and *Ri*Le$^{a/b}$BP to HMOs. (a-c) ITC analysis of *Rh*LNBBP to selected HMOs. **(d-i)** Reference and blank corrected sensograms illustrating binding of selected HMOs to *Ri*Le$^{a/b}$BP. **(j-k)** One binding model fitted to the binding isotherms from the sensograms in (**d-i**). ITC and SPR experiments were performed as duplicates (n=2, independent experiments).

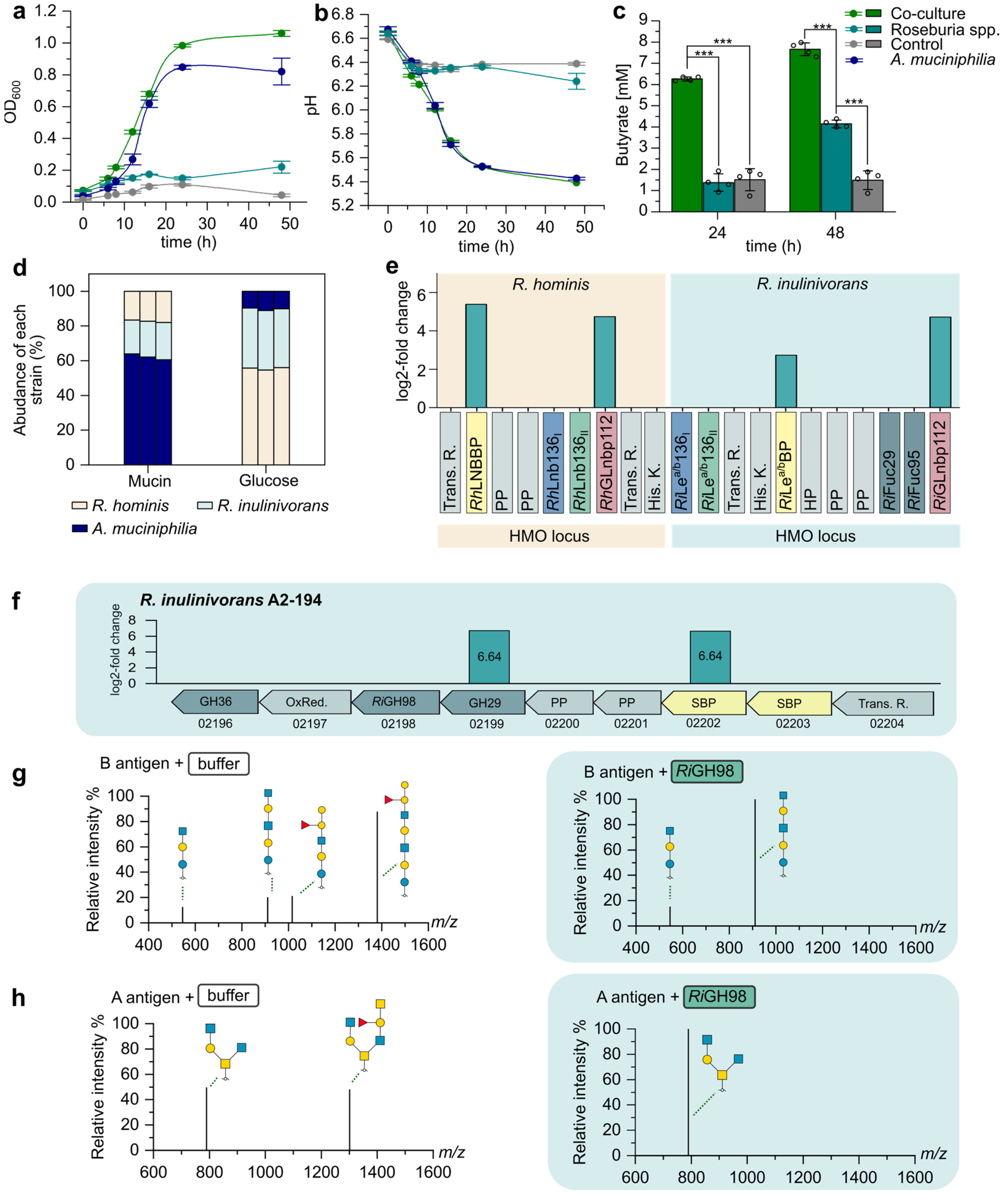

Supplementary Fig. S8: Cross-feeding of *Roseburia* in *A. muciniphilia* co-cultures on mucin. (a-b), Growth of monocultures and co-cultures of *Roseburia* spp. and *A. muciniphila* on mucin. **(c)**, Butyrate in culture supernatants of monocultures and co-cultures as in (a) at 24h and 48h. **(d)**, Relative strain abundance during growth of co-cultures on mucin and glucose at 16 h determined based on MS/MS analyses. **(e)**, Upregulated proteins in the HMOs locus of *R. hominis* and *R. inulinivorans* cells grown in mucin co-culture with *A. muciniphilia* relative to glucose. **(f)**, Upregulated proteins in putative blood group utilization locus of *R. inulinivorans* cells as in (e). **(g-h)** Degradation of Blood group antigen A and B by *Ri*GH98 analysed by nano LC-MS. **(a-c)**, Growth cultures and butyrate quantification were performed in four replicates (n=4 independent experiments) and data are presented as mean values with the error bars representing the standard deviation (SD). (**c**) An unpaired two-tailed Student's *t*-test was used to determine the statistical significance between reached butyrate concentrations, whereby following *p* values where obtained, Co-culture versus *Roseburia* spp: p=8.56 x 10^{-5} (24h), p=1.03 x 10^{-6} (48h); Co-culture versus control p=2.56 x 10^{-4} (24h) and *Roseburia* spp. versus control p=7.68 x 10^{-5} (48h). **(e-h)** Proteomics analyses originate from biological triplicates and nano LC-MS analyses were performed in duplicates. **(c)** Three asterisk (***) indicate a statistically significant difference at a level of p <0.001 **(e),** Protein annotation: transcriptional regulator (Trans. R.); ABC transporter solute binding protein (*Rh*LNBBP (f) and *Ri*$Le^{a/b}$BP (g)); ABC transporter permease protein (PP); hypothetical proteins (HP); Glycoside hydrolase 136 (*Rh*$LNB136_{I}$, *Rh*$LNB136_{II}$ (f) and *Ri*$Le^{a/b}136_{I}$, *Ri*$Le^{a/b}136_{II}$ (g)); Glycoside hydrolase 112 (*Rh*GLnbp 112 (f) and *Ri*GLnbP 112 (g)); Glycoside hydrolase 29 (*Ri*Fuc29 (g)); Glycoside hydrolase 95 (*Ri*Fuc95 (g)) and histidine kinase sensory protein (His. K.). Source data are provided as a Source Data file labelled with the corresponding figure number and panel definition.

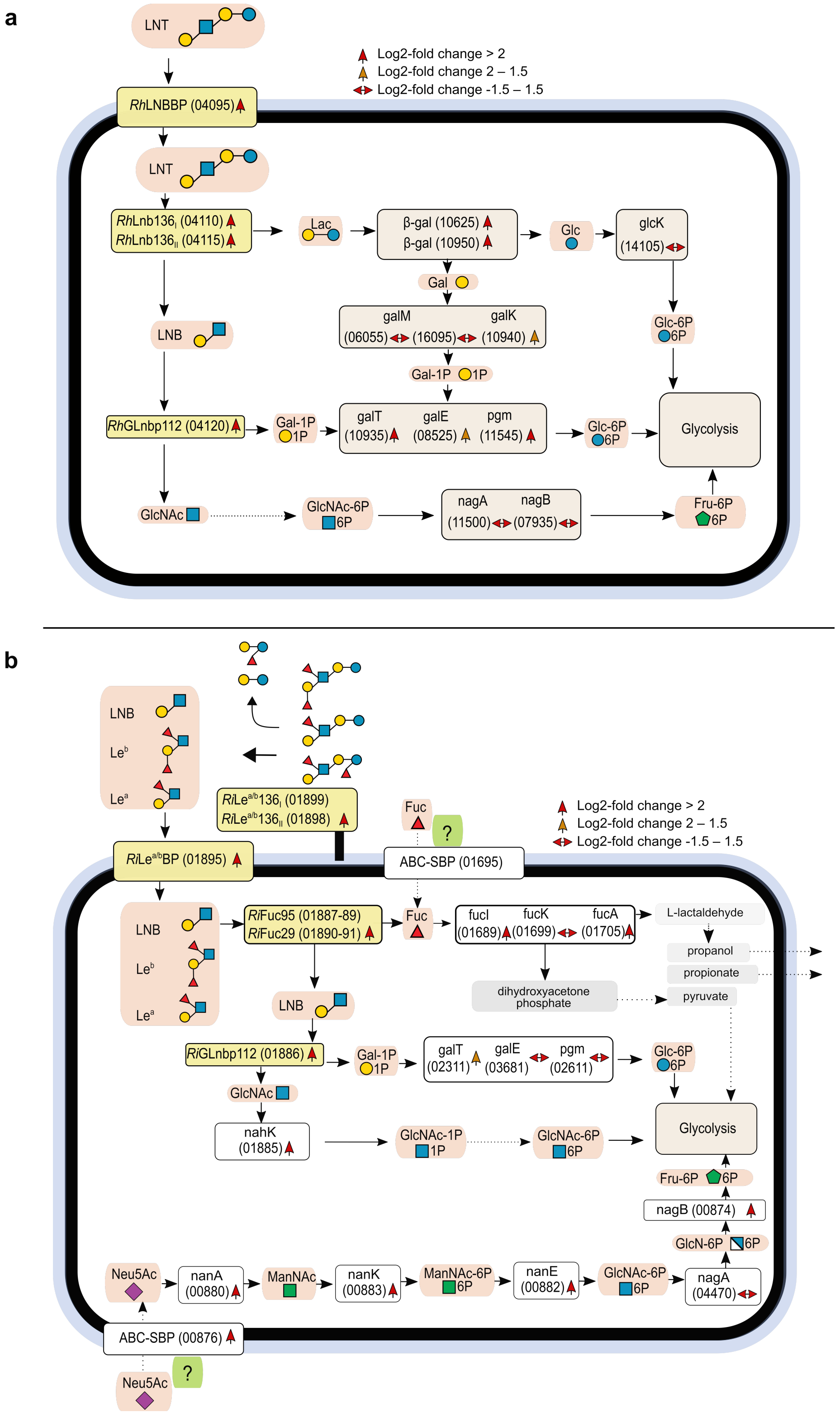

Supplementary Fig. 9: Proposed HMOs core degradation pathways of *R. hominis* and *R. inulinivorans*. (a) Proposed model for LNT utilization in *R. hominis* and **(b)** for fucosylated HMOs utilization in *R. inulinivorans,* based on proteomics data of cells grown on LNT (**a**, *R. hominis*) and HMOs from mother's milk (**b**, *R. inulinivorans*) relative to glucose. Enzymatic steps suggested from literature and detected in proteomics data are indicated in solid arrows, steps suggested by literature but not detected in proteomics data are shown as dotted arrows. Characterized proteins in the present study are highlighted in yellow squares and Locus IDs of *R. hominis* (Rhom_xxxxx) and *R. inulinivorans* (Roseina2194_xxxxx) are abbreviated with the last numbers after the hyphen.
