# Supplementary material for: Butyrate producing colonic Clostridiales metabolise human milk oligosaccharides and cross feed on mucin via conserved pathways
Source: Nat Commun. 2020 Jul 3;11:3285. doi: 10.1038/s41467-020-17075-x (PMC7335108; doi:10.1038/s41467-020-17075-x)
Supplement: Supplementary file 2 — Description of Additional Supplementary Files [file 41467_2020_17075_MOESM2_ESM.docx]

Description of Additional Supplementary Files:

File Name: Supplementary Data 1
Description: LC-MS2 data of *O*-glycan derived oligosaccharide degradation by *Ri*GH98
