# Supplementary material for: Butyrate producing colonic Clostridiales metabolise human milk oligosaccharides and cross feed on mucin via conserved pathways
Source: Nat Commun. 2020 Jul 3;11:3285. doi: 10.1038/s41467-020-17075-x (PMC7335108; doi:10.1038/s41467-020-17075-x)
Supplement: Supplementary file 4 — Reporting Summary [file 41467_2020_17075_MOESM4_ESM.pdf]

nature research

Corresponding author(s): Maher Abou Hachem

Last updated by author(s): June 5, 2020

# Reporting Summary

Nature Research wishes to improve the reproducibility of the work that we publish. This form provides structure for consistency and transparency in reporting. For further information on Nature Research policies, see our Editorial Policies and the Editorial Policy Checklist.

## Statistics

For all statistical analyses, confirm that the following items are present in the figure legend, table legend, main text, or Methods section.

| n/a | Confirmed | |
|---|---|---|
| [ ] | [x] | The exact sample size (*n*) for each experimental group/condition, given as a discrete number and unit of measurement |
| [ ] | [x] | A statement on whether measurements were taken from distinct samples or whether the same sample was measured repeatedly |
| [ ] | [x] | The statistical test(s) used AND whether they are one- or two-sided<br>*Only common tests should be described solely by name; describe more complex techniques in the Methods section.* |
| [x] | [ ] | A description of all covariates tested |
| [x] | [ ] | A description of any assumptions or corrections, such as tests of normality and adjustment for multiple comparisons |
| [ ] | [x] | A full description of the statistical parameters including central tendency (e.g. means) or other basic estimates (e.g. regression coefficient) AND variation (e.g. standard deviation) or associated estimates of uncertainty (e.g. confidence intervals) |
| [ ] | [x] | For null hypothesis testing, the test statistic (e.g. *F*, *t*, *r*) with confidence intervals, effect sizes, degrees of freedom and *P* value noted<br>*Give P values as exact values whenever suitable.* |
| [x] | [ ] | For Bayesian analysis, information on the choice of priors and Markov chain Monte Carlo settings |
| [x] | [ ] | For hierarchical and complex designs, identification of the appropriate level for tests and full reporting of outcomes |
| [x] | [ ] | Estimates of effect sizes (e.g. Cohen's *d*, Pearson's *r*), indicating how they were calculated |

*Our web collection on statistics for biologists contains articles on many of the points above.*

## Software and code

Policy information about availability of computer code

| | |
|---|---|
| Data collection | No custom code or software was used to collect data in this study. Proteomics data were collected on a Q-exactive intrument (Thermo Fisher Scientific) run in a data-dependent manner using a 'Top 10' method. |
| Data analysis | Statistic analyses were performed using Origin 2018b and Origin 2019b (OriginLab). Enzyme activity data were fitted to funtions in detail described in the method section using Origin 2018b and Origin 2019b (OriginLab). Proteome Discoverer versions 2.2 & 2.3 were used to process and analyse the raw MS data files and label free quantification. MALDI-TOF MS analyses were performed using Flexanalysis Version 3.3 (Bruker Daltonics). LC-Ms2 glycomic data were processed using Xcalibur software (version 2.0.7, Thermo Scientific). Relative bacterial abundnaces was estimated on unique peptides identified with Unipept version 4.0. ITC data were analyzed using MicroCal Origin software v7.0. DSC data were analyzed with the NanoAnalyse (v. 3.7.5.) software. For protein crystallization data were processed using HKL2000 ( HKL2000_v720) and XDS (Version March 15, 2019). Initial phase calculation, phase improvement, and automated model building were performed using PHENIX (July 2019). Manual model rebuilding and refinement was achieved using Coot and REFMAC5 (v.5.8). Molecular graphics were prepared using PyMOL v.4.4.0 (Schrödinger, LLC, New York) or UCSF Chimera (1.13.1) (University of California, San Francisco). SignalP v.4.1, PSORTb v3.0 TMHMM v.2.0 were used for prediction of signal peptides and transmembrane domains. InterPro and dbCAN2 were used to analyse modular organization using default settings for Gram positive bacteria. Redundancy in biological sequence datasets was reduced using the CD-HIT server (sequence identity cut off = 0.95). Protein sequence alignments were performed using MAFFT (BLOSUM62). Phylogenetic trees were constructed using the MAFFT server, based on the neighbour-joining algorithm, and with bootstraps performed with 1000 replicates. Phylogenetic trees were visualized and tanglegrams constructed using dendroscope (v.3.7.2). Colouring of protein structures according to amino acid sequence conservation was accomplished in UCSF Chimera (1.13.1) , based on protein multiple (structural based) alignments from the PROMALS3D server and by using the in UCSF Chimera implemented AL2CO algorithm. The MEME suite web server was used for amino acid sequence motif discovery and evaluation. Protein structures were compared using the Dali server (http://ekhidna2.biocenter.helsinki.fi/dali/) (PMID: 27131377) and the molecular interface between ErLnb136I and ErLnb136II was analysed (solvent inaccessible interface, Gibbs energy) via the PDBePISA server (https://www.ebi.ac.uk/pdbe/pisa/). All softwares used in this study are commercial available and the version of each software is additional stated inthe method section. |

For computing the distance matrix of the principle component analysis presented in figure 5 the function stringdistmatrix with the 'osa' method from R was used. The stringdistmatrix function is part of the R-projects stringdist package, available at GitHub (https://github.com/markvanderloo/stringdist)

For manuscripts utilizing custom algorithms or software that are central to the research but not yet described in published literature, software must be made available to editors and reviewers. We strongly encourage code deposition in a community repository (e.g. GitHub). See the Nature Research guidelines for submitting code & software for further information.

## Data

Policy information about availability of data

All manuscripts must include a data availability statement. This statement should provide the following information, where applicable:

- Accession codes, unique identifiers, or web links for publicly available datasets
- A list of figures that have associated raw data
- A description of any restrictions on data availability

The mass spectrometry proteomics data have been deposited to the ProteomeXchange Consortium via the PRIDE partner repository with the dataset identifier PXD015045. The accession numbers for the atomic coordinates reported in this paper are PDB: 6KQS (Se-Met) (https://doi.org/10.2210/pdb6KQS/pdb) and 6KQT (native), see also Table S6. Mucin glycomics MS/MS data are summarized in Table S9 and raw data files are available upon request. Accession number of the cloned genes are provided in Supplementary Table 8. Data underlying Figs.1a-d, Supplementary Figs. 2, 4c, 5a-f and 8a-c are provided as Source Data files and the reproducibility of representative experiments is indicated in the corresponding figure legends. All other data are available from the corresponding author upon requests.

# Field-specific reporting

Please select the one below that is the best fit for your research. If you are not sure, read the appropriate sections before making your selection.

☒ Life sciences ☐ Behavioural & social sciences ☐ Ecological, evolutionary & environmental sciences

For a reference copy of the document with all sections, see nature.com/documents/nr-reporting-summary-flat.pdf

# Life sciences study design

All studies must disclose on these points even when the disclosure is negative.

| | |
|---|---|
| Sample size | No sample-size calculations have been performed in this study. Biological triplicates were performed in all experiments involving bacteria (growth studies and proteomics studies), except bacterial co culture experiments which were performed in 4 independent biological replicates, according to standard scientific methods. |
| Data exclusions | No data was excluded from the data analysis |
| Replication | Technical and biological replicates are indicated in the respective figure legends. All attempts at replication were successful |
| Randomization | In this study randomization was not applicable as no experimental groups were allocated in this study. |
| Blinding | Blinding was not relevant in this study, which does not include animals of human participants |

# Reporting for specific materials, systems and methods

We require information from authors about some types of materials, experimental systems and methods used in many studies. Here, indicate whether each material, system or method listed is relevant to your study. If you are not sure if a list item applies to your research, read the appropriate section before selecting a response.

## Materials & experimental systems

| n/a | Involved in the study | |
|---|---|---|
| ☒ | ☐ | Antibodies |
| ☒ | ☐ | Eukaryotic cell lines |
| ☒ | ☐ | Palaeontology and archaeology |
| ☒ | ☐ | Animals and other organisms |
| ☒ | ☐ | Human research participants |
| ☒ | ☐ | Clinical data |
| ☒ | ☐ | Dual use research of concern |

## Methods

| n/a | Involved in the study | |
|---|---|---|
| ☒ | ☐ | ChIP-seq |
| ☒ | ☐ | Flow cytometry |
| ☒ | ☐ | MRI-based neuroimaging |
